# Short-Term Effects of PENS versus Dry Needling in Subjects with Unilateral Mechanical Neck Pain and Active Myofascial Trigger Points in Levator Scapulae Muscle: A Randomized Controlled Trial

**DOI:** 10.3390/jcm9061665

**Published:** 2020-06-01

**Authors:** Santiago Garcia-de-Miguel, Daniel Pecos-Martin, Tamara Larroca-Sanz, Beatriz Sanz-de-Vicente, Laura Garcia-Montes, Ruben Fernandez-Matias, Tomas Gallego-Izquierdo

**Affiliations:** 1 Physiotherapy and Pain Group, University of Alcalá, 28871 Madrid, Spain; santiago.garciam@uah.es (S.G.-d.-M.); daniel.pecos@uah.es (D.P.-M.); tomas.gallego@uah.es (T.G.-I.); 2 Department of Physical Therapy, University of Alcalá, 28871 Madrid, Spain; 3 Department of Plastic and Restorative Surgery, University Hospital QuironSalud Madrid, 28223 Madrid, Spain; t.larroca@gmail.com; 4 Center of Sports Medicine of the Agencia Española de Protección de la Salud en el Deporte, 28040 Madrid, Spain; beatriz.sanz87@gmail.com; 5 Department of Physical Therapy, General University Hospital Gregorio Marañón, 28007 Madrid, Spain; laugarm@gmail.com; 6 Research Institute of Physiotherapy and Pain, University of Alcala, 28805 Madrid, Spain; 7 Research Unit, Hospital Universitario Fundacion Alcorcon, 28922 Madrid, Spain

**Keywords:** neck pain, PENS, dry needling, myofascial trigger point, physiotherapy

## Abstract

Procedures such as dry needling (DN) or percutaneous electrical nerve stimulation (PENS) are commonly proposed for the treatment of myofascial trigger points (MTrP). The aim of the present study is to investigate if PENS is more effective than DN in the short term in subjects with mechanical neck pain. This was an evaluator-blinded randomized controlled trial. Subjects were recruited through announcements and randomly allocated into DN or PENS groups. Pain intensity, disability, pressure pain threshold (PPT), range of motion (ROM), and side-bending strength were measured. The analyses included mixed-model analyses of variance and pairwise comparisons with Bonferroni correction. The final sample was composed of 44 subjects (22 per group). Both groups showed improvements in pain intensity (η_p_^2^ = 0.62; *p* < 0.01), disability (η_p_^2^ = 0.74; *p* < 0.01), PPT (η_p_^2^ = 0.79; *p* < 0.01), and strength (η_p_^2^ = 0.37; *p* < 0.01). The PENS group showed greater improvements in disability (mean difference, 3.27; 95% CI, 0.27–6.27) and PPT (mean difference, 0.88–1.35; *p* < 0.01). Mixed results were obtained for ROM. PENS seems to produce greater improvements in PPT and disability in the short term.

## 1. Introduction

Dry needling (DN) is a therapeutic intervention in which thin needles are used to penetrate the skin to affect the underlying connective tissue and neuromuscular system without the use of any medication [1]. DN techniques can be sorted according to the depth that the needle is inserted [2]. Although in recent years it has been associated with other musculoskeletal problems, most commonly, DN is designed for the treatment of myofascial trigger points (MTrPs) [3,4]. An MTrP is a discrete, hyperirritable nodule in a taut band of skeletal muscle which is palpable and tender during physical examination [5]. A MTrP may be related to motor dysfunction, fatigue, weakness of the affected muscle, and restricted range of mobility [6]. These hyperirritable nodules can be classified as active, in which case the MTrP is clinically associated with spontaneous pain and, when palpated, reproduces a patient’s familiar pain. They may also be latent MTrPs. Latent MTrPs do not produce spontaneous pain unless they are encouraged [6,7]. DN has been shown to increase the pressure pain threshold (PPT) [8] and range of movement (ROM) [9]; to improve function [10]; and to decrease pain in patients with patellofemoral pain syndrome [10], hip osteoarthritis [11], low-back pain [12], and piriformis syndrome [13].

The possible physiological effects of DN are still not completely understood [14]. It is proposed that DN influences pain by affecting the biochemical environment of the MTrP, reducing the concentration of substance P and calcitonin gene-related peptide surrounding an MTrP [14,15] as well as increasing beta-endorphin in local tissue. In addition, DN would appear to activate different inhibitory pain pathways [16].

The dry needling technique is often non-standardized and may include adjunct treatments, such as electrotherapy. The joint application of DN and electrotherapy has been given numerous names, including electroacupuncture [17], intramuscular electrical stimulation [18], or electrical dry needling [19]. These electric stimulation techniques include the passage of an electrical current through the needle [20]. One of the most utilized techniques to treat MTrPs is percutaneous electrical nerve stimulation (PENS) [20]. PENS is the application of a low-intensity current through an acupuncture needle [19,21]. Although the neuroplasticity processes involved in its effect are not yet well understood, it is a technique of electroacupuncture used to modulate pain processing in some chronic pain conditions, such as myofascial pain syndromes and chronic tensional headache [22,23]. One of the possible mechanisms involved in its effectiveness is a decrease in the excitatory nervous system and a stimulation of the inhibitory nervous system [23,24]. The electrostimulation causes a reduction in the excitability of the motor cortex as well as a decrease in the facilitation of transmission in corticospinal neurons [25]. PENS application to MTrPs would stimulate muscle nociceptors, which activates the endogenous antinociceptive system [21,26]. Several forms of application have been described depending on where the needles are inserted [19,20,27]. 

Current therapeutic interventions for myofascial pain aim to disrupt the maintenance of a contracted state within the MTrP by inhibiting spinal facilitation mechanisms and by increasing blood perfusion to the muscle region [20]. Interventions for treating myofascial pain include dry needling and electrotherapeutic modalities such as PENS [9,20]. These procedures are often used in a clinical setting, and they have been recommended for myofascial pain treatment [1]. The use of DN intervention with electrical stimulation has shown to be effective in the treatment of low-back pain [28] and thoracic spine pain [29]. To our knowledge, no previous studies have investigated whether the PENS application is more effective when a pair of needle electrodes are applied into the MTrP zone in subjects with neck pain. We expect that the local application of PENS electric current to the MTrP is more effective than DN.

The aim of our study was to investigate if PENS application via insertion of a pair of needles into the MTrP of the levator scapulae muscle is more effective that DN for treating neck pain in the short term.

## 2. Experimental Section

### 2.1. Study Design

This randomized controlled trial was conducted according to the recommendations of the Consolidated Standards of Reporting Trials (CONSORT) statement [30].

The protocol of the study was registered at clinicaltrials.gov (NCT03025230). Ethical approval was obtained from the Ethical Committee of the University of Alcala (Madrid, Spain, M2012/19/20130409). The study was conducted according to the Declaration of Helsinki.

### 2.2. Subjects

A convenience sample of subjects with neck pain was recruited through announcements at the city of Alcala. Patients were asked for their medical history to obtain information regarding some of the inclusion and exclusion criteria.

The inclusion criteria were age 18 years or older, nontraumatic unilateral mechanical neck pain of three or more months of duration, using a computer 9 or more hours per week, and presenting an active MTrP in the levator scapulae muscle on the painful side.

The exclusion criteria were radiated pain towards the arm, psychological disorders (mood and psychotic disorders such as schizophrenia, depression, and anxiety disorders), whiplash injury, previous surgical procedures in the cervical spine or shoulder, neuropathic symptoms, received physiotherapy treatment in the previous month, taking anticoagulant or antiplatelet drugs, fear of needles, and any other contraindication to dry needling procedures.

All the subjects signed a consent form before participating in the study. The following demographic data were collected: sex, age, height, weight, body mass index, symptomatic side, and hours per week of computer use. 

### 2.3. Sample Size

The sample size calculation was conducted with the software GPower 3.0.1 (Universität Düsseldorf, Düsseldorf, Germany). It was based on the time-by-group interaction of a 2-by-4 mixed model analysis of variance (ANOVA). The effect size was estimated to be 0.25 with a correlation between repeated measures of 0.50, sphericity correction of 0.75, 90% power, and α value of 0.05. According to the sample size calculation, 36 subjects needed to be recruited. Assuming a maximum dropout rate of 20%, the final sample size was composed of 44 subjects (22 per group).

### 2.4. Measurements

All measurements were carried out in the Faculty of Physiotherapy of the University of Alcala. Measurements were made by a physiotherapist with more than 10 years of experience who was blinded regarding treatment group allocation. Patients were scheduled in a specific time so as not to coincide and were told not to speak with the assessor about his/her clinical status or received treatment.

Pain intensity, pressure pain threshold (PPT), side-bending strength, and range of movement were registered at baseline, posttreatment, 48 h after intervention, and at one-week follow-up. Disability was recorded at baseline and at one-week follow-up.

#### 2.4.1. Pain and Disability

Pain intensity at the moment of measurement was measured with a visual analogue scale (VAS), where 0 represents no pain and 10 represents the worst pain imaginable. The VAS has been shown to be a valid and reliable tool (intraclass correlation coefficient (ICC) 0.71–0.99) [31,32].

Degree of disability related to the cervical spine was measured with the Neck Disability Index (NDI) questionnaire that was transculturally adapted from English into Spanish (Spain) in 2010. The NDI has shown good reliability (ICC 0.98) [33]. The questionnaire points range from 0 (no disability) to 50 (maximum degree of disability). 

#### 2.4.2. Myofascial Trigger Point Diagnosis

The presence of active MTrP in the levator scapulae muscle was tested with the patient lying on the contralateral side before the assignment to interventions, as active MTrP presence was an inclusion criterion. An active MTrP was manually diagnosed if these criteria were present: taut band, hypersensitive spot, local twitch response, and reproduction of the patient’s symptoms [34,35,36]. The levator scapulae is located at the floor of the posterior cervical triangle and commonly develops MTrPs in a central area at the angle of the neck, where the muscle emerges from beneath the anterior border of the upper trapezius muscle. The levator scapulae taut band and MTrP can be palpated with a cross-fiber flat palpation. The local twitch response, which is a local involuntary spontaneous contraction of the muscle evoked when an MTrP is mechanically stimulated, can be produced in the levator scapulae muscle by cross-fiber flat palpation perpendicular to the muscle fiber direction. 

After the active MTrP was identified, the physiotherapist marked its location with a permanent and indelible marker and measured the distance from it to the mastoid apophysis with a caliper [37]. Patients were told not to clean the mark during the entire study period. In addition, the distance between the mastoid apophysis (bone reference) and MTrP was measured to ensure that follow-up measurements were all taken at the same point.

#### 2.4.3. Range of Motion of the Cervical Spine

Range of motion of the cervical spine was measured in a sitting position with a cervical goniometer system (CROM; Performance Attainment Associates, St Paul, MN). The CROM is based on thee goniometers and a magnet system; this tool has been shown to be valid and to have good reliability in healthy and painful subjects (ICC > 0.90) [38].

The following movements were measured: flexion, extension, right and left rotation, and right and left side-bending. Three measurements were taken for each movement, and their mean was used for statistical analysis.

#### 2.4.4. Pressure Pain Threshold

Pressure pain threshold was measured at the location of the active MTrP in the levator scapulae muscle. It was measured with a manual algometer (Wagner Force Dial, Model FDK 20, Wagner Instruments, Burien, WA, USA), which has a 1 cm^2^ head that records pressure in kg/cm^2^. This procedure has shown good reliability in patients with neck pain (ICC = 0.76–0.97) [39].

The pressure was increased by 1 kg per second, and the subject was told to indicate when their sensation changed from pressure to pain. Three measurements were taken with a 30-s rest period between them. The mean of the three measurements was used for statistical analysis.

#### 2.4.5. Side-Bending Strength

The strength measurements were performed with a handheld dynamometer (Model microFET 2^TM^, Hoggan Health Industries Inc., West Jordan, UT, USA), which has been shown to have good reliability in the measurement of cervical side-bending strength (ICC = 0.87–0.95) [40].

The patient was placed lying supine with knees flexed and relaxed in a wedge. The dynamometer was placed on the temporal bone of the painful side. The patient was told to push their head towards the dynamometer and the physiotherapist resisted the movement, so the patient produced an isometric contraction for 5 seconds. Three measurements were taken with a 30-second rest between them, and their mean was used for statistical analysis.

### 2.5. Interventions

Subjects were randomly allocated into intervention groups based on a randomization sequence generated with the software Epidat 3.1 (Conselleria de Sanidade, Xunta de Galicia, Galicia, Spain) prior to the beginning of the study. The allocation was concealed with sequentially numbered sealed envelopes. The group allocation was revealed to the therapist after the initial assessment when the therapist opened the envelope without telling the patient what treatment he/she was going to receive. Before MTrP dry needling, the physiotherapist performed handwashing before putting on nitrile gloves to disinfect the treatment area with cotton and skin antiseptic. Participants were told to maintain their activity level and exercise practice as usual, but they were told not to use analgesic or anti-inflammatory drugs during the experimental period.

#### 2.5.1. Dry Needling

**Figure 1:**
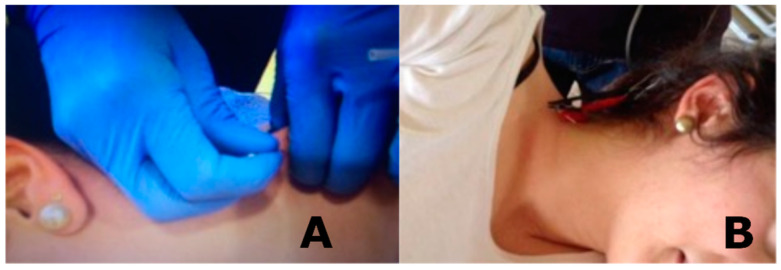
Applied interventions: (**A**) dry needling and (**B**) percutaneous electrical nerve stimulation.

The control group was treated with deep DN (Figure 1) with a needle with a 2.5-cm length and 0.25-cm diameter. The technique was performed in the location of the active MTrP.

Patients were placed lying on the contralateral side. The physiotherapist firmly held the levator scapulae muscle in a pincer grasp to precisely locate the MTrP between his first and second fingers. The therapist directed the needle in an anteroposterior direction towards his finger. Then, the needle was directed upward across the muscle mass as it was held by the fingers to avoid any possibility of penetrating the lung and of causing a pneumothorax [41]. The needle was moved up and down within the muscle, using a “fast-in and fast-out” technique as described by Hong [42]. Needle insertions were repeated 8 to 10 times [43]. After the needling procedure, the therapist compressed the point of needling with cotton wool for 90 seconds.

#### 2.5.2. Percutaneous Electrical Nerve Stimulation

The experimental group was treated with PENS (Figure 1) with the patient placed in the same position as the control group. The experimental group received the same DN protocol in the levator scapulae muscle, but two needles were inserted in the MTrP. After needle insertions were repeated 8 to 10 times, the needles were kept in the muscle. The needles were separated by 1.5 cm [7]

An electrostimulation system was connected to the needles with two crocodile electrical clamps. PENS was applied immediately after, using a portable Transcutaneous Electrical Nerve Stimulation (TENS) (Model P82&S82, Enraf-Nonius, Rotterdam, Netherlands) with an analgesic rectangular, biphasic, and asymmetric current at a 2-Hz frequency with a pulse width of 100 µs [44].

TENS intensity was selected according to the patient’s tolerance and never exceeded 3 mA to avoid tissue injury. For establishing patient’s tolerance, the patient was told before treatment started to tell the therapist when the TENS intensity was so uncomfortable that he or she could not stay at that level of intensity for 1 minute. The TENS was left connected for 20 minutes. After the PENS procedure, the therapist compressed the point of needling with cotton wool for 90 seconds.

### 2.6. Statistical Analysis

Data normality was evaluated with the Shapiro–Wilk test. For the descriptive analysis of quantitative variables, the mean and standard deviation (SD) were used. For categorical variables, the absolute frequencies and percentages were calculated. The homogeneity of the groups was analyzed with the Student’s *t*-test and the chi-squared test [45].

For the differences between groups in pain intensity, PPT, strength, flexion, and extension range of motion, 2-by-4 mixed ANOVAs were conducted with time (baseline, post-treatment, 48 h, and 1 week) as the within-subjects’ factor and treatment (DN and PENS) as the between-subjects’ factor. For the differences between groups in disability, a 2-by-2 mixed model ANOVA was conducted with time (baseline and 1 week) as the within-subjects’ factor and treatment as the between-subjects’ factor. For the differences between groups in cervical rotation and side-bending range of motion, 2-by-2-by-4 mixed ANOVAs were conducted with time and side (painful and nonpainful) as within-subjects’ factors and treatment as the between-subjects factor. Post hoc pairwise comparisons were analyzed using the Student’s *t*-test with Bonferroni correction [45].

The partial eta squared (η_p_^2^) was used as an estimator of the effect size of the main effects and interactions of the ANOVAs [45].

All the analyses were conducted using the statistical software SPSS V.22 (SPSS Inc., Chicago, IL, USA). An α level of 0.05 and 95% confidence intervals (CI) were assumed for all analyses.

## 3. Results

**Figure 2:**
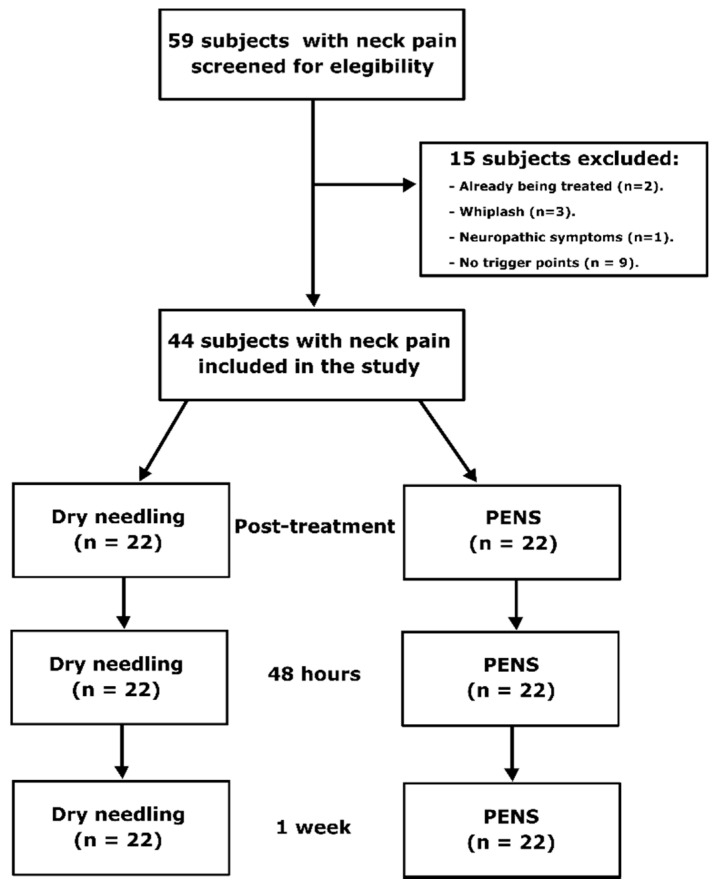
Flow diagram of subjects. PENS, percutaneous electrical nerve stimulation.

The final sample was composed of 44 subjects (Figure 2), 22 subjects in the DN group (mean age, 25.45; SD, 8.53) and 22 subjects in the PENS group (mean age, 24.14; SD, 9.39). Demographic and baseline characteristics of the subjects are presented in Table 1.

### 3.1. Pain and Disability

The 2-by-4 mixed ANOVA for pain intensity revealed a significant main effect for time (F = 68.00; *p* < 0.01; η_p_^2^ = 0.62) but not for group (F = 1.33; *p* = 0.26; η_p_^2^ = 0.03). There was a nonsignificant time-by-group interaction (F = 0.37; *p* = 0.67; η_p_^2^ = 0.01).

The 2-by-2 mixed ANOVA for disability revealed a significant main effect for time (F = 121.67; *p* < 0.01; η_p_^2^ = 0.74) but not for group (F = 0.12; *p* = 0.73; η_p_^2^ = 0.003). There was a significant time-by-group interaction (F = 4.87; *p* = 0.03; η_p_^2^ = 0.10). Post hoc comparisons are presented in Table 2.

### 3.2. Pressure Pain Threshold

The 2-by-2 mixed ANOVA for PPT revealed a significant main effect for time (F = 154.97; *p* < 0.01; η_p_^2^ = 0.79) and group (F = 60.89; *p* < 0.01; η_p_^2^ = 0.59) as well as a significant time-by-group interaction (F = 31.07; *p* < 0.01; η_p_^2^ = 0.43). Post hoc comparisons are presented in Table 3.

### 3.3. Side-Bending Strength

The 2-by-4 mixed ANOVA for strength revealed a significant main effect for time (F = 24.84; *p* < 0.01; η_p_^2^ = 0.37) but not for group (F = 0.29; *p* = 0.59; η_p_^2^ = 0.01). There was a nonsignificant time-by-group interaction (F = 0.55; *p* = 0.65; η_p_^2^ = 0.01). Post hoc comparisons are presented in Table 3.

### 3.4. Range of Motion of the Cervical Spine

The 2-by-4 mixed ANOVA for cervical flexion range of motion revealed a significant main effect for time (F = 9.94; *p* < 0.01; η_p_^2^ = 0.19) and group (F = 4.16; *p* = 0.048; η_p_^2^ = 0.09) as well as a significant time-by-group interaction (F = 3.25; *p* = 0.046; η_p_^2^ = 0.07). Post hoc comparisons are presented in Table 4.

The 2-by-4 mixed ANOVA for cervical extension range of motion revealed a significant main effect for time (F = 10.55; *p* < 0.01; η_p_^2^ = 0.20) but not for group (F = 2.44; *p* = 0.07; η_p_^2^ = 0.06). There was a nonsignificant time-by-group interaction (F = 0.29; *p* = 0.60; η_p_^2^ = 0.01). Post hoc comparisons are presented in Table 4.

The 2-by-2-by-4 mixed ANOVA for cervical rotation range of motion revealed a significant main effect for time (F = 17.53; *p* < 0.01; η_p_^2^ = 0.57) but not for group (F = 4.07; *p* = 0.05; η_p_^2^ = 0.09) or side (F = 1.19; *p* = 0.28; η_p_^2^ = 0.03). There were no significant time-by-group (F = 0.09; *p* = 0.97; η_p_^2^ = 0.01), time-by-side (F = 1.34; *p* = 0.27; η_p_^2^ = 0.09), group-by-side (F = 0.03; *p* = 0.87; η_p_^2^ = 0.001), or time-by-group-by-side (F = 1.35; *p* = 0.27; η_p_^2^ = 0.09) interactions. Post hoc comparisons are presented in Table 5.

The 2-by-2-by-4 mixed ANOVA for cervical side-bending range of motion revealed a significant main effect for time (F = 27.02; *p* < 0.01; η_p_^2^ = 0.39) but not for group (F = 0.95; *p* = 0.34; η_p_^2^ = 0.02) or side (F = 2.19; *p* = 0.15; η_p_^2^ = 0.05). There were a significant time-by-side (F = 5.33; *p* < 0.01; η_p_^2^ = 0.11) and time-by-group-by-side (F = 4.70; *p* = 0.01; η_p_^2^ = 0.10) interactions. There were no significant time-by-group (F = 0.33; *p* = 0.78; η_p_^2^ = 0.01) or group-by-side (F < 0.001; *p* = 0.99; η_p_^2^ < 0.001) interactions. Post hoc comparisons are presented in Table 6.

## 4. Discussion

### 4.1. Pain and Disability

Both groups showed a decrease in pain at all follow-up measurement points without differences between groups. These improvements agree with a recent meta-analysis that suggest PENS improves pain [46]. Posttreatment decreases in VAS were near the minimum clinically important difference (MCID) of 2 cm, whereas improvements in pain intensity at 48-hour and 1-week follow-up were above the MCID [47]. This nonsignificant small but greater decrease in pain intensity at 48-hour and 1-week follow-up in comparison to posttreatment measures could be due to the aggressivity of needling therapies, which could limit the therapeutic potential at posttreatment follow-up, as has been observed in previous studies [48].

Leon-Hernandez et al. [21] found that the addition of percutaneous TENS to DN significantly reduced cervical pain at posttreatment follow-up in comparison with DN alone. However, that difference was not maintained at 72 h follow-up. These differences between groups at posttreatment follow-up disagree with the results of the present study. This discrepancy could be because Leon-Hernandez et al. [21] performed the DN technique until two local twitch responses were evoked, while in the present study, needle insertions were repeated 8 to 10 times, so the dose of DN treatment in the present study could be greater. It has been observed that performance of DN until no twitch response is evoked produces more post-needling soreness in comparison to when no twitch response is evoked. However, there seems to be no difference when few twitch responses are evoked in comparison to DN until no twitch response is evoked [49]. Therefore, DN with more twitch responses produces a greater mechanical stimulus, which could increase its therapeutic potential without increasing post-needling soreness. This could explain the differences between the results of Leon-Hernandez et al. [21] and those of the present study.

The absence of between-group differences in the present study could be because of the compression applied at the end of the needling procedures. It has been shown that ischemic compression reduces post-needling soreness until 48 h after the procedure [50]. Therefore, the compression of 90 seconds applied in the present study just after the interventions could have decreased the possible potential differences between DN and PENS. 

A greater improvement in the degree of disability measured with NDI in the PENS group in comparison with the DN group at one-week follow-up was found. Leon-Hernandez et al. [21] did not find a difference between groups at 72 h follow-up. It may be necessary to allow more follow-up time to appreciate the differences between electrical treatments and DN, as was shown in the present study. This improvement in disability could be explained by the greater improvements in PPT in the PENS group in comparison with the DN group observed in the present study. The decrease in muscle mechanosensitivity could cause the subject to feel less pain during active movements that contract or elongate the levator scapulae muscle during the studied week, thus diminishing their perception of neck disability. Furthermore, previous studies have found an association between neck disability and fear of movement [51]. In some cases, patients with neck pain may develop an avoidance-hypervigilance attitude, which could trigger a vicious cycle in which the patient is affected both physically and psychologically, leading to deconditioning [52]. Kinesiophobia is greater among patients with neck disability [53]. These patients believe that movement will cause reinjury and additional pain [53]. Over time, these patients can develop physical deconditioning, which limits the adequate execution of a movement or exercise and leads to more sedentary behavior [54]. In the present study, PENS showed a greater improvement in the degree of disability. It is probable that disability improvement will have major implications for a change of health-related quality of life and will enhance physical activity and work capacity in subjects with neck pain [55].

### 4.2. Pressure Pain Threshold

Both groups showed an increase in the pressure pain threshold in all measurement follow-ups. All the increases in PPT were above the minimum detectable change (MDC) of 0.48 kg/cm^2^ reported by Walton et al. [39] in subjects with neck pain.

Zaeifar et al. [48] found an increase in PPT after DN therapy but not at posttreatment follow-up. This could be because Zaeifar et al. [48] did not realize an ischemic compression after DN, which could help to increase PPT just after DN, and thus diminishing post-needling soreness, as previous studies have shown [50].

The PENS group showed a statistically significant greater increase in PPT in comparison with DN in all follow-ups. These results agree with those of Leon-Hernandez et al. [21] with percutaneous TENS. The greater improvements in the PENS group could be because electrostimulation produced a “wash-out effect” thorough muscle contractions induced by the electrical current, which could help to wash out algogenic substances, thus decreasing mechanosensitivity and increasing local PPT of the muscle [56]. However, it should be pointed out that other studies have also found an increase in PPT in distal locations with DN, suggesting a central nervous system implication in the induced hypoalgesia [57]. The absence of a distal measurement point in the present study prevents us from knowing if the between-group differences observed could be due to either a central or local component.

### 4.3. Strength

Improvement in side-bending strength in both groups at 48-hour and 1-week follow-ups was found but not immediately posttreatment. The absence of posttreatment improvements could be because more time is needed for changes induced by DN or PENS in the levator scapulae muscle to lead to an increase in strength. Other studies have also found an increase in cervical side-bending strength with DN [58], and a recent meta-analysis showed that DN could produce improvements in cervical strength at one-month follow-up [59].

The improvements observed at 48-hour and 1-week follow-ups were below the MDC of 3–4 kg reported by Versteegh et al. [40]. However, in that study, the strength was self-measured by the subjects, who usually are not experienced in this kind of procedure, thus decreasing the reliability of their strength measurements. In contrast, in the present study, the measures were recorded by an external physiotherapist, which could in turn lead to an increase in the reliability of the procedure, thus decreasing the MDC. However, as we did not evaluate the reliability of our procedure, we cannot ensure that the observed differences are not due to measurement errors.

There were no between-group differences at any follow-up measurement points. This could be because of the small follow-up period, so we cannot know if some differences would be observed at mid-term and long-term follow-ups.

### 4.4. Cervical Range of Movement

There was only a significant increase in cervical flexion range of movement in the PENS group at all follow-ups. Although the PENS group showed greater improvement in cervical flexion than the DN group at all measurement points, there was only a statistically significant between-group difference at posttreatment follow-up. Both groups increased cervical extension range of motion without differences between them. Cervical flexion has been shown to increase the mechanical stress of the nervous system compared to cervical extension [60]; thus, differences between groups in cervical flexion but not cervical extension could be because the effectiveness of PENS is mediated by responses upon the central nervous system [21,26].

Both groups showed an increase in cervical range of motion without any between-group differences. The PENS group showed an increase in cervical side-bending towards painful and nonpainful sides, whereas the DN group only showed an increase in cervical side-bending towards the nonpainful side. The levator scapulae muscle elongates with contralateral side-bending [61]. The DN group had less side-bending motion towards the nonpainful side compared to the painful side at baseline, and the motion towards the painful side was within the normal range [62]. It could be that most effectiveness of DN is local to the muscle which, along with the baseline differences, could explain why the DN group only improved towards the nonpainful side. Furthermore, both groups had raw values of side-bending within the normal range at all follow-ups, thus making it difficult to draw conclusions about the superiority of one treatment over another. Although there was also a between-side difference in rotation range of motion at the baseline in the DN group, according to previous data, both sides had less range of motion than healthy people [62], so this absence of an upper limit constraint could have prevented the same between-side differences results being obtained in cervical side-bending.

Some of these results agree with those obtained by Leon-Hernandez et al. [21]. However, the inconsistencies discussed above within the results of this study precludes us from knowing if PENS produces greater improvements than DN in cervical range of motion. 

### 4.5. Limitations

The principal limitation of the current study is the small follow-up time, which could have contributed to the absence of between-group differences in some outcomes. It would be interesting to conduct future research with longer follow-up periods to clarify if these differences might exist.

The inclusion of a compression after the needling procedures in both groups could have also conditioned the results observed, hiding some between-group differences. Furthermore, two needles were used in the PENS group and only one in the DN group, which may be another factor that could limit the conclusions of the study about the effectiveness of PENS in comparison with DN.

Cervical side-bending is produced by other muscles than the levator scapulae, and there is no way to isolate the contraction of the levator scapulae muscle to measure its strength. This precludes us from knowing if the treatments produced an improvement in the capability of the levator scapulae muscle to produce strength.

Finally, we did not include a placebo group so placebo effects cannot be discarded. All treatments were delivered in a context that includes social and physical cues, verbal suggestions, and treatment history. This context is actively interpreted by the subject and can elicit expectations, memories, and emotions that can influence health-related outcomes [63]. A large part of the overall therapeutic response to treatments may be due to the treatment context rather than the specific treatment itself. In this study, the application time of the PENS treatment was greater than that of the DN group. This time difference could lead to greater positive expectations in patients who received the PENS treatment, and thus, the differences in effectiveness may have been because of a greater placebo effect [64]. Finally, although a recent meta-analysis has showed that PENS is better than sham PENS for decreasing pain intensity [46], we cannot exclude some placebo effect in our study results.

## 5. Conclusions

Percutaneous electrical nerve stimulation seems to produce greater improvements in mechanosensitivity and disability than DN at short-term follow-up. However, there seem to be no between-group differences regarding pain intensity and side-bending strength and there were inconsistencies regarding range of motion. More research is needed with mid-term and long-term follow-ups to evaluate the possible differences between these treatments over time.

## Figures and Tables

**Table 1:** Baseline characteristics of the subjects (*n* = 44).

Characteristic, Mean (SD)	DN (*n* = 22)	PENS (*n* = 22)	*p*-Value
Age, years	25.45 (8.53)	24.14 (9.39)	0.63
Height, cm	171.73 (8.86)	171.59 (6.60)	0.95
Weight, kg	65.42 (10.79)	63.49 (8.58)	0.51
BMI, kg/m^2^	21.64 (2.67)	21.60 (2.02)	0.95
Computer Use, hours/week	15.77 (10.82)	16.92 (7.84)	0.69
MTrP Distance, cm	8.12 (1.07)	8.08 (1.13)	0.93
VAS, cm	5.01 (1.52)	4.56 (1.73)	0.36
NDI	19.64 (7.32)	22.09 (9.75)	0.35
PPT, kg/cm^2^	1.98 (0.56)	2.11 (0.52)	0.43
Strength, N	145.20 (28.70)	152.40 (32.20)	0.44
Flexion, Degrees	44.83 (9.85)	44.59 (11.25)	0.94
Extension, Degrees	50.72 (9.72)	52.81 (13.90)	0.57
Rotation, Degrees			
Painful Side	58.21 (15.46)	61.59 (10.50)	0.40
Nonpainful Side	52.18 (12.78)	59.47 (14.47)	0.08
Side Bending, Degrees			
Painful Side	38.59 (5.81)	38.50 (5.07)	0.96
Nonpainful Side	34.30 (5.31)	37.45 (3.65)	0.03
Sex, *n* (%)			0.53
Male	9 (40.9)	7 (31.8)	
Female	13 (59.1)	15 (68.2)	
Painful Side, *n* (%)			0.07
Right	16 (72.7)	10 (45.5)	
Left	6 (27.3)	12 (54.5)	

Abbreviations: SD, standard deviation; DN, dry needling; PENS, percutaneous electrical nerve stimulation; BMI, body mass index; h, hours; w, week; MTrP, myofascial trigger point; VAS, visual analogue scale; NDI, Neck Disability Index; PPT, pressure pain threshold.

**Table 2:** Pain and disability differences within- and between-group.

Variable	Baseline	Post-Treatment	48 Hours	1 Week
VAS, cm				
DN, Mean (SD)	5.01 (1.52)	3.30 (1.70)	2.28 (1.58)	2.26 (1.55)
PENS, Mean (SD)	4.56 (1.73)	2.62 (2.11)	2.04 (1.69)	1.71 (1.29)
Within-Group Differences from Baseline, Mean (95% CI)				
DN		1.71 (1.02, 2.40) ^‡^	2.73 (1.83, 3.64) ^‡^	2.76 (1.86, 3.65) ^‡^
PENS		1.94 (1.25, 2.63) ^‡^	2.52 (1.61, 3.42) ^‡^	2.85 (1.96, 3.74) ^‡^
Between-Group Differences, Mean (95% CI)		−0.23 (−0.94, 0.48)	0.22 (−0.72, 1.15)	−0.09 (−1.01, 0.83)
NDI				
DN, Mean (SD)	19.64 (7.32)	-	-	13.09 (7.45)
PENS, Mean (SD)	22.09 (9.75)	-	-	12.27 (8.20)
Within-Group Differences from Baseline, Mean (95% CI)				
DN				6.55 (4.43, 8.66) ^‡^
PENS				9.82 (7.70, 11.94) ^‡^
Between-Group Differences, Mean (95% CI)				−3.27 (−6.27, −0.27) ^†^

^†^ Statistically significant (*p* < 0.05), ^‡^ Statistically significant (*p* < 0.01). Abbreviations: VAS, visual analogue scale; DN, dry needling; SD, standard deviation; PENS, percutaneous electrical nerve stimulation; CI, confidence interval; NDI, Neck Disability Index.

**Table 3:** Pressure pain threshold and strength differences within- and between-group.

Variable	Baseline	Post-Treatment	48 Hours	1 Week
PPT, kg/cm^2^				
DN, Mean (SD)	1.98 (0.56)	2.71 (0.52)	2.72 (0.46)	2.70 (0.52)
PENS, Mean (SD)	2.11 (0.52)	3.71 (0.46)	3.84 (0.43)	4.18 (0.46)
Within-Group Differences from Baseline, Mean (95% CI)				
DN		0.73 (0.44, 1.01) ^‡^	0.74 (0.47, 1.01) ^‡^	0.72 (0.39, 1.04) ^‡^
PENS		1.61 (1.32, 1.89) ^‡^	1.73 (1.45, 2.00) ^‡^	2.06 (1.74, 2.39) ^‡^
Between-Group Differences, Mean (95% CI)		0.88 (0.58, 1.17) ^‡^	0.99 (0.71, 1.27) ^‡^	1.35 (1.01, 1.68) ^‡^
Strength, N				
DN, Mean (SD)	145.30 (28.70)	155.30 (28.80)	163.20 (33.00)	173.00 (37.10)
PENS, Mean (SD)	152.40 (32.20)	162.70 (32.70)	167.10 (32.90)	173.90 (33.60)
Within-Group Differences from Baseline, Mean (95% CI)				
DN		10.00 (−2.80, 22.80)	18.00 (6.20, 29.80) ^‡^	27.80 (15.30, 40.30) ^‡^
PENS		10.20 (−2.60, 23.00)	14.70 (2.90, 26.50) ^‡^	21.50 (9.00, 34.00) ^‡^
Between-Group Differences, Mean (95% CI)		0.20 (−13.00, 13.40)	−3.30 (−15.40, 8.90)	−6.30 (−19.20, 6.60)

^†^ Statistically significant (*p* < 0.05), ^‡^ Statistically significant (*p* < 0.01). Abbreviations: PPT, pressure pain threshold; DN, dry needling; SD, standard deviation; PENS, percutaneous electrical nerve stimulation; CI, confidence interval.

**Table 4:** Cervical flexion and extension range of movement differences within- and between-group.

Variable	Baseline	Post-Treatment	48 Hours	1 Week
Cervical Flexion, Degrees				
DN, Mean (SD)	44.83 (9.85)	45.90 (8.61)	48.36 (9.31)	47.27 (8.41)
PENS, Mean (SD)	44.59 (11.25)	52.95 (7.50)	54.73 (6.39)	51.27 (7.59)
Within-Group Differences from Baseline, Mean (95% CI)				
DN		1.08 (−4.25, 6.41)	3.53 (−3.35, 10.32)	2.44 (−3.74, 8.62)
PENS		8.36 (3.04, 13.69) ^‡^	10.14 (3.35, 16.92) ^‡^	6.68 (0.50, 12.86) ^†^
Between-Group Differences, Mean (95% CI)		7.29 (1.79, 12.78) ^†^	6.60 (−0.39, 13.60)	4.24 (−2.13, 10.61)
Cervical Extension, Degrees				
DN, Mean (SD)	50.72 (9.72)	53.50 (9.86)	57.32 (7.49)	58.05 (7.95)
PENS, Mean (SD)	52.81 (13.90)	58.41 (10.14)	56.64 (11.37)	57.68 (11.26)
Within-Group Differences from Baseline, Mean (95% CI)				
DN		2.78 (−1.09, 6.65)	6.60 (1.00, 12.19) ^†^	7.33 (2.11, 12.54) ^‡^
PENS		5.59 (1.72, 9.46) ^‡^	3.82 (−1.77, 9.41)	4.86 (−0.36, 10.08)
Between-Group Differences, Mean (95% CI)		2.81 (−1.18, 6.80)	−2.78 (−8.54, 2.98)	−2.46 (−2.92, 7.84)

^†^ Statistically significant (*p* < 0.05), ^‡^ Statistically significant (*p* < 0.01). Abbreviations: DN, dry needling; SD, standard deviation; PENS, percutaneous electrical nerve stimulation; CI, confidence interval.

**Table 5:** Cervical rotation range of movement differences within- and between-group.

Variable	Baseline	Post-Treatment	48 Hours	1 Week
DN, Mean (SD)				
Painful Side	58.21 (14.46)	64.41 (8.92)	68.09 (9.32)	66.50 (8.95)
Nonpainful Side	52.18 (12.78)	64.5 (8.95)	66.27 (12.69)	66.14 (10.67)
PENS, Mean (SD)				
Painful Side	61.59 (10.50)	69.55 (9.38)	71.50 (8.02)	70.55 (7.32)
Nonpainful Side	59.48 (14.47)	67.68 (9.36)	70.41 (9.02)	69.68 (9.52)
Within-Group Differences from Baseline, Mean (95% CI)				
DN				
Painful Side		6.20 (−0.84, 13.25)	9.88 (2.15, 17.62) ^‡^	8.29 (0.59, 16.00) ^†^
Nonpainful Side		12.32 (6.85, 17.80) ^‡^	14.10 (7.52, 20.68) ^‡^	13.96 (7.18, 20.74) ^‡^
PENS				
Painful Side		7.96 (0.91, 15.00) ^†^	9.91 (2.18, 17.64) ^‡^	8.96 (1.25, 16.66) ^†^
Nonpainful Side		8.21 (2.73, 13.68) ^‡^	10.93 (4.35, 17.51) ^‡^	10.21 (3.43, 16.98) ^‡^
Between-Group Differences, Mean (95% CI)				
Painful Side		1.75 (−5.51, 9.01)	0.03 (−7.95, 8.00)	0.66 (−7.28, 8.61)
Nonpainful Side		−4.12 (−9.76, 1.52)	−3.16 (−9.95, 3.62)	−3.76 (−10.74, 3.23)

^†^ Statistically significant (*p* < 0.05), ^‡^ Statistically significant (*p* < 0.01). Abbreviations: DN, dry needling; SD, standard deviation; PENS, percutaneous electrical nerve stimulation; CI, confidence interval.

**Table 6:** Cervical side-bending range of movement differences within- and between-group.

Variable	Baseline	Post-Treatment	48 Hours	1 Week
DN, Mean (SD)				
Painful Side	38.59 (5.81)	40.09 (4.00)	39.73 (5.68)	40.91 (4.05)
Nonpainful Side	34.30 (5.31)	41.00 (4.92)	40.45 (4.93)	40.50 (5.32)
PENS, Mean (SD)				
Painful Side	38.50 (5.07)	42.27 (5.19)	41.81 (4.15)	41.41 (5.61)
Nonpainful Side	37.45 (3.65)	41.82 (5.24)	40.27 (6.02)	41.36 (5.52)
Within-Group Differences from Baseline, Mean (95% CI)				
DN				
Painful Side		1.50 (−0.67, 3.68)	1.14 (−1.89, 4.16)	2.32 (−0.43, 5.07)
Nonpainful Side		6.70 (4.15, 9.26) ^‡^	6.16 (2.96, 9.35) ^‡^	6.20 (3.16, 9.25) ^‡^
PENS				
Painful Side		3.77 (1.60, 5.95) ^‡^	3.32 (0.29, 6.34) ^†^	2.91 (0.16, 5.66) ^†^
Nonpainful Side		4.36 (1.81, 6.92) ^‡^	2.82 (−0.38, 6.01)	3.91 (0.87, 6.95) ^‡^
Between-Group Differences, Mean (95% CI)				
Painful Side		2.27 (0.03, 4.51) ^†^	2.18 (−0.94, 5.30)	0.59 (−2.24, 3.42)
Nonpainful Side		−2.34 (−4.97, 0.29)	−3.34 (−6.63, −0.05) ^†^	−2.29 (−5.43, 0.84)

^†^ Statistically significant (*p* < 0.05), ^‡^ Statistically significant (*p* < 0.01). Abbreviations: DN, dry needling; SD, standard deviation; PENS, percutaneous electrical nerve stimulation; CI, confidence interval.
